# Attitudes and barriers towards conducting research amongst primary care physicians in Bahrain: a cross-sectional study

**DOI:** 10.1186/s12875-019-0911-1

**Published:** 2019-01-26

**Authors:** Abeer J. Khalaf, Aysha I. Aljowder, Meead J. Buhamaid, Mona F. Alansari, Ghufran A. Jassim

**Affiliations:** 1 Family Practice Residency Program, Ministry of Health, PO Box 12, Juffair, Bahrain; 2 Family Medicine, Royal College of Surgeons in Ireland-Medical University in Bahrain, PO Box 15503, Adliya, Bahrain

**Keywords:** Research, Attitude, Barriers, Primary care, Physicians

## Abstract

**Background:**

Research in primary care is essential for disease diagnosis, management and prevention in relation to the individuals, families and the community. This research aims to study the attitude of primary care physicians towards conducting research in Bahrain and to identify the main barriers encountered during research.

**Methods:**

A cross-sectional study was conducted amongst 200 randomly selected primary care physicians registered in Ministry of Health affiliated primary healthcare centers in Bahrain. A self-administered validated questionnaire was adopted and used for data collection. Research data was analyzed using the Statistical Package for Social Sciences (SPSS) version 23.

**Results:**

Primary care physicians had a positive attitude towards conducting research with a total mean score (SD) of 4.47(0.65) (on a scale from 1 to 5 with higher scores indicating more positive attitudes). The total mean score (SD) for barriers encountered by physicians during research was 3.34 (0.80). Insufficient research allotted time (76.5%), insufficient financial support (63%), lack of financial incentives (51%) and lack of statistical support (50%) were major barriers. Physicians designation and board certificate were significantly associated with attitudes and barriers towards research (*P*-value < 0.05).

**Conclusion:**

The majority or primary care physicians had positive attitudes towards conducting research. The major difficulties faced by physicians in conducting research are: Insufficient research allotted time, lack of financial incentives and inadequate statistical support. The study addressed a gap in building research capacity which should be embraced by many institutions through partnership and collaboration.

**Electronic supplementary material:**

The online version of this article (10.1186/s12875-019-0911-1) contains supplementary material, which is available to authorized users.

## Background

Primary care is the first contact and principal point of continuing care for patients in the healthcare system [1]. General practice is the cornerstone of successful primary health care, which underpins population health outcomes and is key to ensuring we have a high-quality, equitable and sustainable health system into the future [2].

Research in primary care aims largely to seek better understanding of disease diagnosis, management and prevention, and to evaluate the effectiveness and efficiency of health care practices and health policies [3]. As a result, there is an urgency to upscale primary care research to translate this evidence into regular clinical practice [4]. Thus, it is important for primary care physicians to be equipped with the necessary skills to conduct research and to interpret, critically appraise and apply research.

Several studies have reported primary care physicians’ positive attitude towards conducting research in practice [5, 6]. The majority of primary care physicians appreciated the importance of conducting research and believed research was relevant to practice [7, 8]. However, in Bahrain, very limited research has been conducted in this field. Only one study touched on the importance of Evidence Based Medicine (EBM), research methodology and critical appraisal skills. It showed that 42% of primary care physicians have attended EBM workshops while 61% claim to use EBM in their practice [9]. Although this does not reflect the attitudes of physicians towards research specifically, it sheds lights on the utilization of physicians on research-based evidence in their practice.

Despite the notion that the majority of primary care physicians in different parts of the world view research as a fundamental component of their practice, few studies have explored the barriers towards conducting research in the Arabian Gulf region as opposed to a wealth of information in the West [10, 11]. A study in Oman outlined the barriers towards conducting research amongst healthcare professionals. One third of participants in the study found that financial support (32.3%), research allotted time (31.8%) and financial incentives (30.3%) are the main barriers. Interestingly, the participants who had undergraduate research training had a more positive attitude towards research and experienced less barriers compared to those who did not receive training [10].

In Bahrain, the attitudes and barriers of physicians towards conducting research have not been studied in depth as there is scarcity of research describing this topic. In order to increase sustainable research capacity in developing countries such as Bahrain, it is important to understand the context of the local health research landscape, and to gain insight into factors that influence research activity both positively and negatively. This study will impact policy makers and research-training directorates to recognize if further training is needed and identify ways of overcoming barriers to conducting research by primary health care physicians. 

## Methods

A cross-sectional study amongst 337 primary care physicians registered in Ministry of Health affiliated primary healthcare centers was conducted in Bahrain. Slovin’s formula was used to calculate the sample size needed with a confidence level of 95% and a margin of error of 0.05 [12]. A total of 200 participants were randomly selected via a computer software program. Verbal consent was acquired from those selected. Those who declined to participate in the study or were missing during the two-week data collection period were replaced by the person next on the list.

We adopted a self-administered questionnaire that had been previously used in a large survey of clinicians in Oman and had been assessed for face validity by experts in the field [10]. We piloted the questionnaire on 20 participants after which minor changes were carried out to clarify and simplify language. Ethics approval was obtained from the research committee at the Ministry of Health.

The questionnaire had two different components. The first section aimed to collect socio-demographic data *(gender, age, nationality, years of practice, designation, specialty board certification).* The second section aimed to assess primary care physicians’ attitude and identify possible barriers towards conducting research, using a Likert’s scale 1–5. Higher scores indicated a more positive attitude and greater barriers (Additional file 1).

Research data were analyzed using the Statistical Package for Social Sciences (SPSS) version 23. Descriptive statistics of frequencies, percentages and mean were presented. The mean for each element in the attitudes and barriers sections was calculated separately for each of the 200 participants. The overall physicians’ attitude and barriers was then determined by calculating the total mean for each section using the pre-calculated means. The highest possible total mean was 5 and the lowest was 1. Higher scores indicated more positive attitude and barriers. The relationship between demographic data and participants’ attitude and barriers towards research was then studied using the total mean scores via the ANOVA and t-test measures.

## Results

### Socio-demographic data

Two hundred physicians consented to participation. Of the total, 82% (*n* = 163) were Bahrainis, 68% (*n* = 136) were females and the mean age (SD) was 43(10.06). The mean years of practice of participants was 16(9.56). Moreover, 42% (*n* = 84) were family physician consultants, 32% (*n* = 64) were family physician specialists and 26% (*n* = 52) were general practitioners. In addition, 166 participants (83%) were Arab and Irish board certified (Table 1).

**Table 1:** Demographic Data

		n	%
Age groups	*< 40*	81	45.0%
	*40–50*	54	30.0%
	*> 50*	45	25.0%
Gender	*Male*	64	32.0%
	*Female*	136	68.0%
Nationality	*Bahraini*	163	81.9%
	*Non-Bahraini*	36	18.1%
Years of Practice Grouping	*< 10*	58	32.8%
	*10–20*	72	40.7%
	*> 20*	47	26.6%
Designation	*GP*	52	26.0%
	*Family Physician Specialist*	64	32.0%
	*Family Physician Consultant*	84	42.0%
Specialty Board Certificate	*Yes*	166	83.0%
	*No*	34	17.0%

### Physicians’ attitude and barriers towards conducting research

Results showed that primary care physicians had a positive attitude towards conducting research with a total mean score (SD) of 4.47(0.65) (on a scale from 1 to 5 with higher scores indicating more positive attitudes) (Table 2). A more positive attitude was noted with more senior physician designation (*p* = 0.003) and with being specialty board certified (*p* = 0.009) (Table 3).

**Table 2:** Attitude towards conducting research

	Mean	SD
Promotes critical thinking	4.48	.80
Improves patient care	4.48	.81
Helps in promotion	4.49	.85
Helps professional enhancement	4.55	.71
Helps to change health policy	4.34	.98
Overall Attitude	4.47	.65

When assessing the possible barriers that participants’ are most likely to encounter, the total mean score (SD) was 3.34(0.80) (Table 4). It appeared that insufficient research allotted time (76.5%), insufficient financial support (63%), lack of financial incentives (51%) and lack of statistical support (50%) were the major barriers. Factors that showed significant association with barriers encountered by physicians towards conducting research were designation (*p* < 0.001) and specialty board certificate (*p* = 0.011) (Table 5). The reliability of the questionnaire in our study was tested and yielded a Cronbach’s alpha of > 70%.

**Table 3:** Relationship between demographic data and attitude toward conducting research

		Attitude		Test	*P*-value	95% CI
		Mean	SD			
Age groups	< 40	4.49	.59	ANOVA	0.098	(4.36,4.62)
	40–50	4.61	.51			(4.48,4.75)
	> 50	4.34	.81			(4.10,4.58)
Gender	Male	4.49	.64	T-test	0.772	(4.33,4.65)
	Female	4.46	.66			(4.35,4.57)
Nationality	Bahraini	4.47	.63	T-test	0.961	(4.37,4.56)
	Non-Bahraini	4.47	.77			(4.21,4.73)
Years of Practice Grouping	< 10	4.54	.47	ANOVA	0.497	(4.41,4.66)
	10–20	4.49	.64			(4.34,4.64)
	> 20	4.40	.74			(4.18,4.61)
Designation	GP	4.21	.86	ANOVA	0.003	(3.97,4.45)
	Family Physician Specialist	4.53	.46			(4.41,4.64)
	Family Physician Consultant	4.58	.58			(4.45,4.71)
Specialty Board Certificate	Yes	4.54	.55	T-test	0.009	(4.46,4.63)
	No	4.09	.92			(3.77,4.42)

Higher mean score indicates a more positive attitude

**Table 4:** Barriers Towards Conducting Research

	Mean	SD
Lack of research training and skills	3.26	1.27
Lack of statistical support	3.48	1.26
Lack of mentorship and teamwork	3.23	1.24
Insufficient financial support	3.76	1.42
Technical and logistic support like computer and internet not easily available	2.83	1.41
Lack of self-interest and motivation	2.51	1.29
Lack of communication and linkages with other institutions	3.33	1.22
Lack of financial incentives	3.42	1.39
Overall Barriers	3.34	.80

**Table 5:** Relationship between demographic data and barriers toward conducting research

		Barriers		Test	*P*-value	95% CI
		Mean	SD			
Age groups	< 40	3.40	.75	ANOVA	0.602	(3.24,3.57)
	40–50	3.34	.80			(3.12,3.56)
	> 50	3.25	.81			(3.01,3.50)
Gender	Male	3.21	.84	T-test	0.123	(3.00,3.42)
	Female	3.40	.78			(3.27,3.53)
Nationality	Bahraini	3.39	.78	T-test	0.082	(3.27,3.51)
	Non-Bahraini	3.14	.87			(2.84,3.43)
Years of Practice Grouping	< 10	3.40	.75	ANOVA	0.818	(3.20,3.60)
	10–20	3.41	.79			(3.23,3.60)
	> 20	3.32	.80			(3.09,3.56)
Designation	GP	3.02	.89	ANOVA	0.000	(2.78,3.27)
	Family Physician Resident	3.31	.78			(3.11,3.50)
	Family Physician Consultant	3.56	.69			(3.41,3.72)
Specialty Board Certificate	Yes	3.42	.75	T-test	0.011	(3.31,3.53)
	No	2.96	.96			(2.62,3.29)

Higher mean score indicates higher barriers encountered by physicians

## Discussion

Research in primary care has a significant role in enhancing physicians’ clinical knowledge and improving patients’ overall care. This study provides an important overview of the attitudes towards research held by primary care physicians as well as the barriers that influence research capacity and production. It is likely that other developing countries in the region face the same challenges.

Overall, physicians had positive attitudes towards conducting research in terms of promoting critical thinking, improving patient care, helping in promotion and professional enhancement, in addition to helping shape healthcare policies. However, results from this study find that this alone may not be enough to increase the culture and productivity of research. Results found that insufficient research allotted time, lack of financial incentives and inadequate statistical support were the major difficulties prevents physicians from fully engaging in research activities. This was consistent with the findings of other studies conducted in the region [8, 10]. This is also in accordance with international literature. For example, a German study found that 85% of general practitioners had a positive attitude toward conducting research [5] with clinical and administrative assignments being the most recognized barriers [5]. Another study conducted in United Kingdom found that 90% of general practitioners appreciated the importance of research and believed it to be essential, while 68% used research to aid their clinical practice [7]. Also, literature has shown that financial incentives and infrastructural support are essential for conducting research [11, 13].

Positive attitudes toward research was significantly associated with more senior physician position and being awarded specialty board certificate. Designations of primary care physicians in Bahrain range from General practitioner (graduate from medical university) and is the most junior position, to family physician resident who is trained in the family residency program following completion of medical school and the most senior is family physician consultant who had completed family residency training, has at least four years of clinical work experience and had been awarded the specialty board certificate. This finding is expected as family residency program involves advanced training in research methodology and statistics. Further, the same group of board certified physicians with senior designations reported significant amount of barriers to conducting research. This could be attributed to the fact that research output is essential for these physicians either as part of their training or as part of their clinical promotion. The same finding was not reported in the literature and this could be explained in view of the unique promotion system in our clinical setting.

Research can seem daunting and eccentric to doctors who would rather be users of research than undertake research themselves. But the question is why it is important for family physicians to conduct research? Research in primary care is important because doing research makes us better critics and translators of evidence [14, 15].

In the context of clinical focused practices, personal motivation is a fundamental facilitator for increased research activity. In order to enhance and promote a medical research environment and to improve research productivity among primary care physicians, various requirements need to be considered at many levels. At the academic level, supporting training programs and providing more educational and practical training opportunities for students, residents and physicians. Also, raising awareness on the importance of research academically, professionally and scientifically among such populations. Additionally, using the expertise in universities and academic institutions to promote research attitudes and practices and to provide educational support and workshops. A framework should be developed to make the various research questions operational and to point out the gaps in our research. At a higher level, policy makers should reconsider budget allocation for research and balancing the working hours of physicians to allow for more research time. Nevertheless, this will not be accomplished without appropriate research infrastructure in order to improve the outcome of the undertaken research. Thus, there is a need for an advanced research center with data collection and statistical services in place in order to conduct research efficiently.

One of the major strengths of our study is the random sampling of primary care physicians from all those registered in Bahrain. Another advantage of our study is the well-structured, pre-designed questionnaire used in our data collection with high reliability (Cronbach’s alpha > 70%).

As with all research, there are some unavoidable limitations. This study can be generalized to the primary health care physicians affiliated with the Ministry of Health only, but not those in the private sector as we did not include them in our sample. Secondly, there was limited local literature to compare our results to which makes them difficult to reinforce.

## Conclusion

The majority or primary care physicians had positive attitudes towards conducting research. The major difficulties faced by physicians in conducting research are: Insufficient research allotted time, lack of financial incentives and inadequate statistical support. The study addressed a gap in building research capacity which should be embraced by many institutions through partnership and collaboration.

## Additional file

Additional file 1: Questionnaire. Brief description of the data: This file contains the survey tool that was used in this study. (DOCX 392 kb)
