# Supplementary material for: Attitudes and barriers towards conducting research amongst primary care physicians in Bahrain: a cross-sectional study
Source: BMC Fam Pract. 2019 Jan 26;20:20. doi: 10.1186/s12875-019-0911-1 (PMC6347740; doi:10.1186/s12875-019-0911-1)
Supplement: Supplementary file 1 — Questionnaire. Brief description of the data: This file contains the survey tool that was used in this study. (DOCX 392 kb) [file 12875_2019_911_MOESM1_ESM.docx]

**Attitude and Barriers Towards Conducting Research Amongst Primary Care Physicians in Bahrain**

**Questionnaire**

**Section A: Demography**

*The first part of the questionnaire aims to collect basic background information on you. Kindly fill out the requested data.*

**Age: _______**

**Gender:** □ Male □ Female

**Nationality**: □ Bahraini □ Non-Bahraini

**Years of practice (including the FPRP years where applicable): _________years**

**Designation:**

□ GP □ Family Physician Resident □ Family Physician Consultant

□ Other specify: _________________

**Highest Educational Degree Attained: (tick more than one if needed)**

□ MBBS /MD

□ Specialty Board Certificate

□ Masters

□ PhD

□ Diploma

□ Other

**Presently involved in any research**: □ Yes □ No

**Conducted a research by your own:** □ Yes □ No

**Previous research training**

**(at least 8 hours of research education):** □ Yes □ No

**Previously published a research:**  □ Yes □ No

**Section B: Attitude & Barriers Towards Conducting Research**

*The first part of this section assesses primary care physician’s perceived value of research within the clinical setting. Please rate the extent to which you believe research is helpful by ticking the appropriate box.*


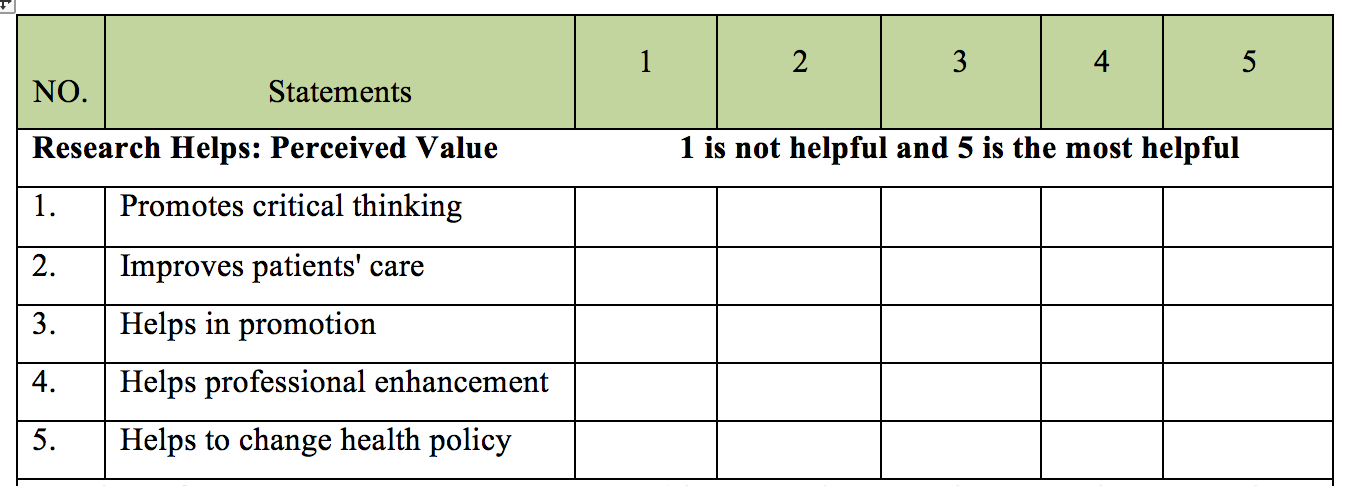


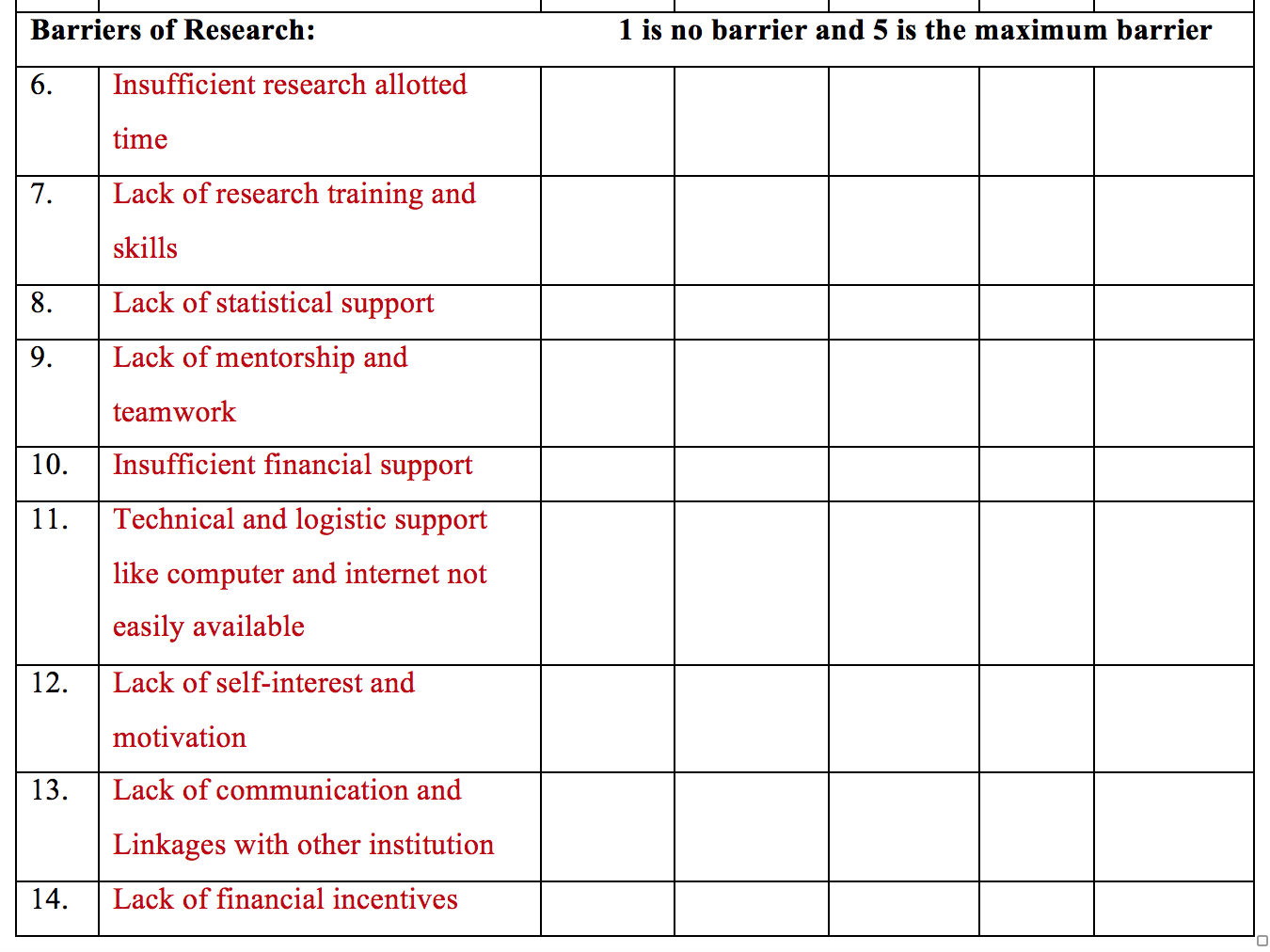

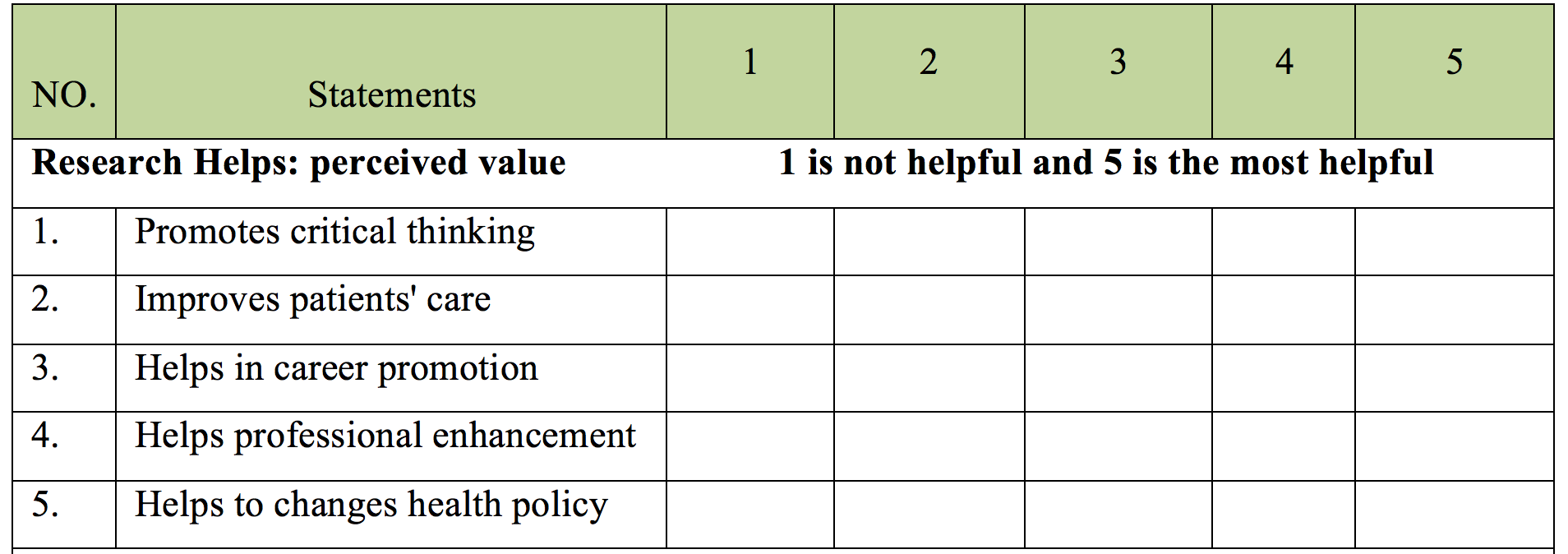
*The second part of this section evaluates the barriers towards conducting research among primary care physicians. Please read each statement below and rate appropriately.*

**Section C: Research self-experience:**

*This last section of the questionnaire aims to study primary care physician’s’ previous experience in conducting research. Please rate your level of experience in each of the following.*

| **No** | **Research Self-Experience** | **1**  **No Experience** | **2** | **3** | **4** | **5**  **Very Experienced** |
| --- | --- | --- | --- | --- | --- | --- |
| 1. | Writing a protocol |  |  |  |  |  |
| 2. | Writing and presenting a research report |  |  |  |  |  |
| 3. | Critically reviewing literature |  |  |  |  |  |
| 4. | Finding relevant literature |  |  |  |  |  |
| 5. | Generating research ideas |  |  |  |  |  |
| 6. | Analyzing and interpreting data |  |  |  |  |  |
| 7. | Submitted a research paper for publication |  |  |  |  |  |
| 8. | Applied and received a grant |  |  |  |  |  |
| 9. | Abstract presentation at a conference |  |  |  |  |  |
